# Regional variations and spatial heterogeneity of lumbar CT attenuation are associated with osteoporotic vertebral fracture

**DOI:** 10.3389/fendo.2025.1630371

**Published:** 2025-09-16

**Authors:** Jinhui Cai, Ludan Chen, Long Liu, Jinsheng Yi, Jiaqi Wu, Tingqian Yang, Wensheng Huang, Qingyu Liu

**Affiliations:** Department of Radiology, The Seventh Affiliated Hospital, Sun Yat-sen University, Shenzhen, Guangdong, China

**Keywords:** lumbar vertebrae, osteoporotic vertebral fracture, vertebral trabecular Hounsfield units, regional variations, spatial heterogeneity

## Abstract

**Summary:**

Osteoporotic vertebral fracture (OVF) constitutes a prevalent health concern in the elderly. Reduced vertebral HU values and increased spatial heterogeneity in the L1 and L2 vertebrae were independently associated with OVF. The HU values combined with spatial heterogeneity quantification could be a feasible approach for opportunistic OVF risk assessment.

**Purpose:**

Examine the associations between vertebral Hounsfield units (HU) and osteoporotic vertebral fracture (OVF), with a particular emphasis on regional variations and spatial heterogeneity of vertebral trabeculae.

**Methods:**

The regional (anterior, middle, posterior, superior, inferior) and total HU in L1 and L2 vertebrae were measured, and with spatial distribution quantified through regional HU ratios. Heterogeneity in HU were assessed using interquartile range (IQR) and coefficient of variation (CV). Group differences were analyzed by Mann-Whitney U test and t-test, while multiple comparisons of CT measurements were adjusted using the Benjamini-Hochberg (B-H) method. Logistic regression identified independent factors associated with OVF, and ROC curves evaluated the diagnostic efficacy of vertebral HU for vertebral fracture prediction.

**Results:**

This retrospective case-control study comprising 54 individuals with acute OVF and 108 age- and sex-matched controls. The regional and total HU of L1 and L2 (B-H adjusted p< 0.001) decreased in OVF patients compared to the controls. The OVF patients exhibited higher CV in both L1 and L2, and CV (per 10% increased) were positively associated with increased odds of OVF independent to vertebral HU and T-score (L1: adjusted OR 2.845; 95% CI, 1.076 - 7.524; p= 0.035 and L2: adjusted OR 2.944; 95% CI, 1.246 - 6.955; p= 0.014). ROC revealed moderate predictive accuracy for total vertebral HU (L1: AUC = 0.715; L2: AUC = 0.738), with marginally superior performance in inferior regions (L1: AUC = 0.716; L2: AUC = 0.740).

**Conclusion:**

Reduced vertebral HU values and increased spatial heterogeneity in L1 and L2 vertebrae were associated with OVF, providing valuable references for OVF risk assessment.

## Introduction

Osteoporotic vertebral fracture (OVF) is the most common type of osteoporotic fracture, typically leading to back pain and disability, increasing the medical and socioeconomic burden (1). More importantly, patients exhibit an elevated risk of premature mortality for the first 5 years following an OVF (2, 3). The overall mortality rates within 2-years are shown to increase to 20.61% for men, and 10.48% for women (3). Accurate identification of at-risk populations for OVF is essential for early intervention and the implementation of appropriate treatments to mitigate the risk of future fractures.

Currently, areal bone mineral density (BMD) measured by dual-energy X-ray absorptiometry (DXA), is considered the gold standard for the diagnosis of osteoporosis and the risk assessment method of OVF. A T-score value of -2.5 standard deviations (SD) or lower (T-score ≤ -2.5SD) is indicative of osteoporosis and at high-risk of OVF (4). Although areal BMD derived from DXA can identify at-risk individuals, there is considerable overlap in areal BMD between those who will fracture and those who will not (5), resulting in limited predictive ability for fracture risk, as about 41%-50% of OVF happen in individuals without osteoporosis (6, 7). Furthermore, additional limitations of DXA, including affordability, limited accessibility, and low screening rates, constrain its widespread clinical application (8).

Routine clinical computed tomography (CT) examinations of the chest, abdomen, and/or lumbar spine have been employed for opportunistic osteoporosis screening (9, 10) and vertebral fracture risk assessment (11–14). These scans yield detailed insights into vertebral bone characteristics by rapidly and conveniently quantifying the CT attenuation in Hounsfield Units (HU) of the vertebral trabecular region (15). Nevertheless, the assessment of vertebral trabecular attenuation was usually performed using a two-dimensional methodology, measuring the mean HU of vertebral trabecular bone in just a singular region, typically located at the center of the vertebrae. Previous experimental studies have demonstrated that the intravertebral density and architecture are not uniformly distributed (16, 17). The heterogeneous distribution of density and local variations in the microstructure within the vertebral trabecular bone may influence its strength and associated with fragility vertebral fracture (17–19). Recently, a clinical study (20) has also demonstrated that the HU values of the anterior, middle and posterior regions of the lumbar vertebral body exhibited significant differences from level L1 to L3 across all age groups (20-79 years), with the lowest HU values in the anterior region. However, the association between the regional variations and spatial heterogeneity of lumbar vertebrae as measured by HU values, and vertebral fractures remains inadequately understood.

This work applied a three-dimensional approach to evaluate CT attenuation across various anatomical regions of the L1 and L2 vertebral bodies of the lumbar spine. The primary objective was to investigate the association between vertebral CT attenuation and OVF, with a specific emphasis on regional variations and spatial heterogeneity in CT HU values within the lumbar vertebrae.

## Subjects and methods

### Study population

**Figure 1:**
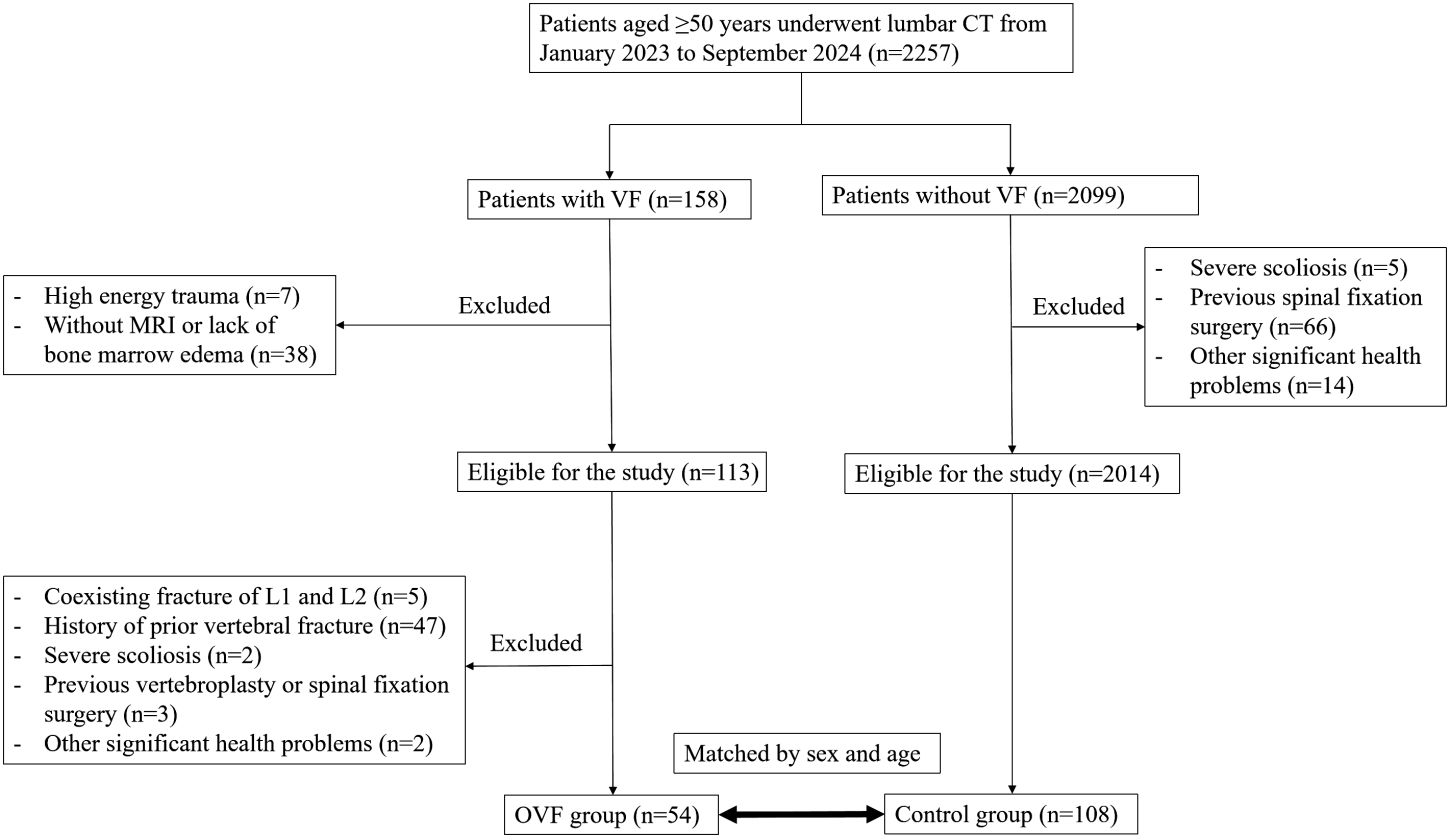
The flowchart for patient screening and selection.

In this case-control study, we conducted a retrospective review of 2,257 consecutive patients aged over 50 years who underwent lumbar CT scans at our institution between January 2023 and September 2024. The patients were categorized into the OVF group or the control group (without OVF) based on the presence or absence of acute vertebral fractures at the time of the CT scans. Inclusion criteria for the OVF group were as follows: i) patients aged over 50 years with at least one acute vertebral fracture occurred from the T1 to L5 vertebrae due to minimal or minor trauma, and ii) evidence of bone marrow edema in the fractured vertebral bodies as demonstrated by spinal MRI. Exclusion criteria included: i) patients with acute vertebral fractures that occurred in both L1-L2 level, as these levels were used to calculate CT attenuation, ii) patients with a history of prior vertebral fracture, vertebroplasty or spinal fixation surgery at any level of T1-L5, iii) the presence of severe scoliosis of the spine, and iv) patients with other significant health problems, for instance, mental illness, severe cardiopulmonary comorbidity, major coagulopathy, or long term used of glucocorticoid. A total of 54 patients were ultimately enrolled in the OVF group for the final analysis. Each subject in the OVF group was matched by sex and age (± 2 years) in a 1:2 ratio to establish the control group (n= 108) (**Figure 1**). Demographic data, including age, sex, weight, height, and body mass index (BMI), were collected for all subjects enrolled in this study. The Institutional Review Board of our hospital granted ethical approval for this study and waived the requirement for written informed consent due to its retrospective design.

### Dual-energy X-ray absorptiometry

DXA measurements were performed using standardized protocols on a high-quality densitometer (GE Lunar Prodigy Pro, GE Healthcare, USA) by experienced technologists under the supervision of a certified densitometrist. The auto-centering routine was implemented to ensure accurate spinal alignment during the scanning process, with a tube voltage of 140/100 kV and a current of 2.5 mA. The L1 to L4 vertebrae were assessed in the anterior-posterior projection (5). Vertebral bodies exhibiting fractures, severe regional structural changes were excluded from the analysis. Patients were excluded if only one vertebra remained after these exclusions. The mean T-score for the lumbar spine was reported, with a threshold for identifying high risk of OVF defined as T-score ≤ −2.5 SD (4).

### CT image acquisition

All lumbar CT scans were performed with a 320-row multi-detector CT scanner (uCT 960+, United Imaging, China) and the scanning parameters were as follows: slice thickness 1 mm, distance 1 mm, tube voltage 120 kV, and automatic milliampere modulation. The CT images were reconstructed using a soft standard kernel (B_SOFT_B) with a pixel matrices size of 512 × 512.

### Imaging measurement procedure

Morphological parameters and HU values of the L1 and L2 vertebrae were evaluated by an experienced radiologist (LD Chen) utilizing free and open-source software (3D Slicer, Version 5.7.0). In instances where the L1 (n= 11) or L2 (n= 6) vertebral bodies were insufficient (e.g., the presence of OVF) to conduct the measurement, an adjacent vertebral body (T12 for L1 or L3 for L2) were substituted to acquire these features.

**Figure 2:**
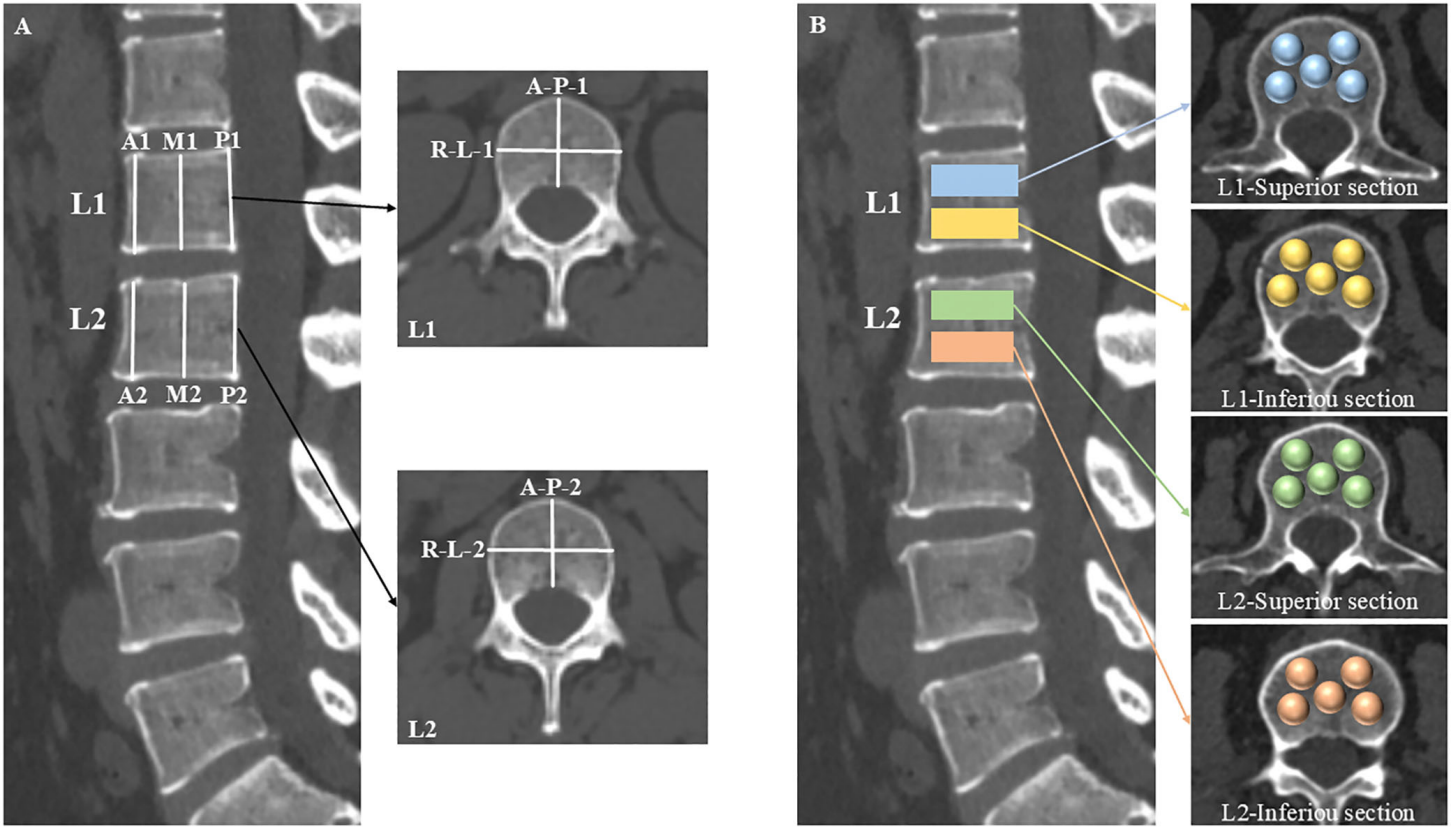
Schematic diagrams of morphology and CT attenuation measurements. The central sagittal plane of the lumbar CT was used to measure the anterior (A1, A2), middle (M1, M2), and posterior (P1, P2) heights of the L1 and L2 vertebrae with a six-point method. The anterior-posterior (A-P-1, A-P-2) and right-left (R-L-1, R-L-2) widths were calculated by their maximum diameters, respectively, in the central axial plane of the L1 and L2 vertebrae **(A)**. The L1 and L2 vertebral bodies were segmented into superior and inferior sections. Within each section, five distinct locations were identified as spherical VOIs for HU values measurements: the right-anterior, left-anterior, central, right-posterior, and left-posterior sub-regions of the vertebral body **(B)**.

The morphometry measurement approach of the L1 and L2 vertebrae were shown in **Figure 2A**. The anterior, middle, and posterior heights of the vertebrae were assessed within the central sagittal plane of the lumbar spine using a six-point method (21), which includes measurements at the four corners of the vertebral body and the midpoints of the endplates. Additionally, the anterior-posterior and right-left widths were determined in the central axial plane of the L1 and L2 vertebrae by calculating the maximum anterior-posterior and right-left diameters.

The L1 and L2 vertebral bodies were segmented into superior and inferior sections, respectively. Within each section, five distinct locations were identified as volumes of interest (VOIs) for HU values measurements: the right-anterior, left-anterior, central, right-posterior, and left-posterior sub-regions of the vertebral body (**Figure 2B**). The VOIs were configured as spheres to facilitate the acquisition of volumetric HU values, deliberately excluding cortical bone and heterogeneous structures such as Schmorl’s nodes, bone islands, and the posterior venous plexus.

### Regional analysis in vertebral CT attenuation

For the regional analysis of HU values within the vertebrae, five distinct anatomical regions were delineated: (i) the anterior region, defined by the mean HU values derived from the right- and left-anterior sub-regions of both the superior and inferior sections; (ii) the middle region, characterized by the mean HU values from the central sub-regions of the superior and inferior sections; (iii) the posterior region, identified by the mean HU values from the right- and left-posterior sub-regions of the superior and inferior sections; (iv) the superior region, determined by the mean HU values of the five sub-regions comprising the superior sections; (v) the inferior region, defined by the mean HU values of the five sub-regions comprising the inferior sections; and (vi) the total vertebral HU value was calculated as the average of the HU values obtained from the ten sub-regions within the vertebrae.

### Spatial distribution and heterogeneity in vertebral CT attenuation

The spatial distribution of HU values within the vertebrae were quantified using four regional ratio-based methods: anterior/middle, anterior/posterior, middle/posterior, and superior/inferior HU ratios. The intravertebral heterogeneity in CT attenuation was assessed based on the ten sub-regional VOIs of the L1 and L2 vertebral bodies, respectively. Two quantitative metrics were employed to measure heterogeneity in HU values: (i) the interquartile range (IQR), defined as the difference between the third quartile (Q3) and the first quartile (Q1) HU values of the ten sub-regions; and (ii) the coefficient of variation (CV), calculated as the standard deviation divided by the mean of the HU values across the ten sub-regions.

### Statistical analyses

The categorical variable of sex was represented as frequencies and percentages, and group differences were analyzed using chi-square tests. Continuous variables were expressed as mean ± standard deviation (SD). For normally distributed variables, comparisons between the OVF and control groups were made using the t-test, while variables with non-normal distribution were analyzed using the Mann-Whitney U test. To account for multiple testing of CT measurements and control the false discovery rate, the p values of these comparisons were adjusted using the Benjamini-Hochberg (B-H) method. Univariate and multivariate logistic regression analyses were employed to identify independent factors associated with OVF. Variables with p values < 0.2 in the univariate model were included in the multivariate analysis, and adjusted odds ratios (OR) with 95% confidence intervals (CIs) were calculated. Receiver Operating Characteristic (ROC) curves for the differential regional or total vertebral HU values of the L1 and L2 vertebrae, as well as the T-score of the lumbar spine, were plotted to evaluate the diagnostic performance for predicting OVF. Statistical significance in the area under the curve (AUC) differences was assessed using the DeLong test. All statistical analyses were performed using the freely available R software (version 4.4.2; https://www.r-project.org), and the statistical significance level was set to < 0.05.

## Results

### Patient characteristics

A total of 162 patients, with ages ranging from 50 to 85 years, were enrolled in this case-control study, and the demographic and clinical features of the patients are presented in **Table 1**. The T-score of lumbar spine was significantly lower in the OVF group compared to the control group (-3.23 ± 1.30 versus -1.98 ± 1.52 SD, p< 0.001), with no significant differences observed in age, sex, and BMI between the two groups (all p> 0.05).

**Table 1:** Comparison of demographic and clinical characteristics between the OVF group and the control group.

Characteristics	OVF (n= 54)	Control (n= 108)	P value
Sex, n (%)			
Female	43 (79.6%)	86 (79.6%)	1.000
Male	11 (20.4%)	22 (20.4%)	
Age (yeas), mean ± SD	65.3 ± 9.2	65.1 ± 9.1	0.932
Height (cm), mean ± SD	155.5 ± 5.9	157.6 ± 5.6	**0.034**
Weight (kg), mean ± SD	58.6 ± 8.9	60.9 ± 6.1	0.084
BMI, mean ± SD	24.2 ± 3.2	24.5 ± 2.1	0.503
Lumbar BMD			
T-score (SD), mean ± SD	-3.23 ± 1.30	-1.98 ± 1.52	**< 0.001**

(OVF, osteoporotic vertebral fracture; BMI, body mass index; BMD, bone mineral density; SD, standard deviation). P value in bold indicated statistical significance.

### Association between CT imaging measures and OVF

**Figure 3:**
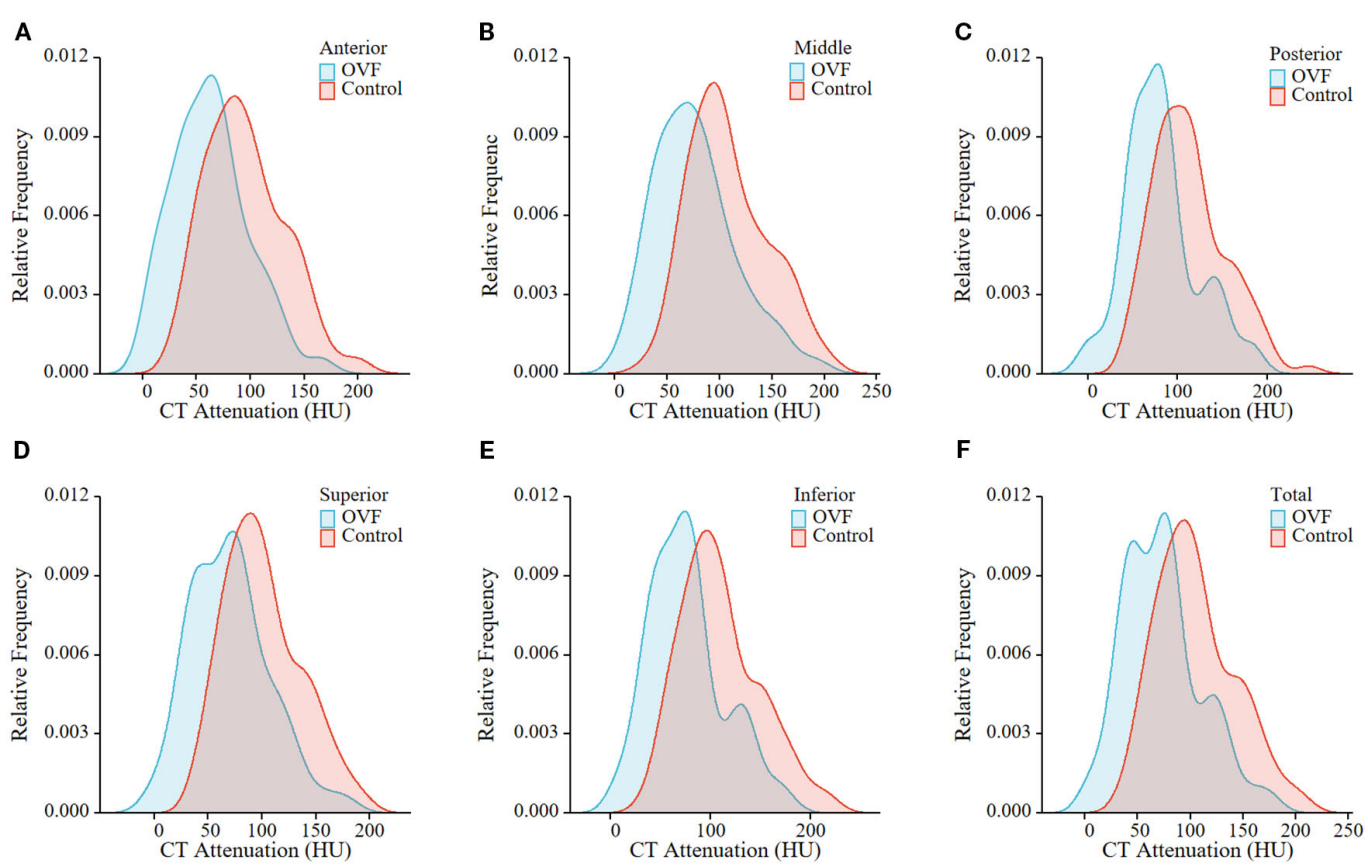
Relative frequency polygon shows CT attenuation of the anterior region **(A)**, middle region **(B)**, posterior region **(C)**, superior region **(D)**, and inferior region **(E)**, and total vertebrae **(F)** of L1 in the individuals with OVF and the controls. (OVF, osteoporotic vertebral fracture; HU, Hounsfield Units).

**Figure 4:**
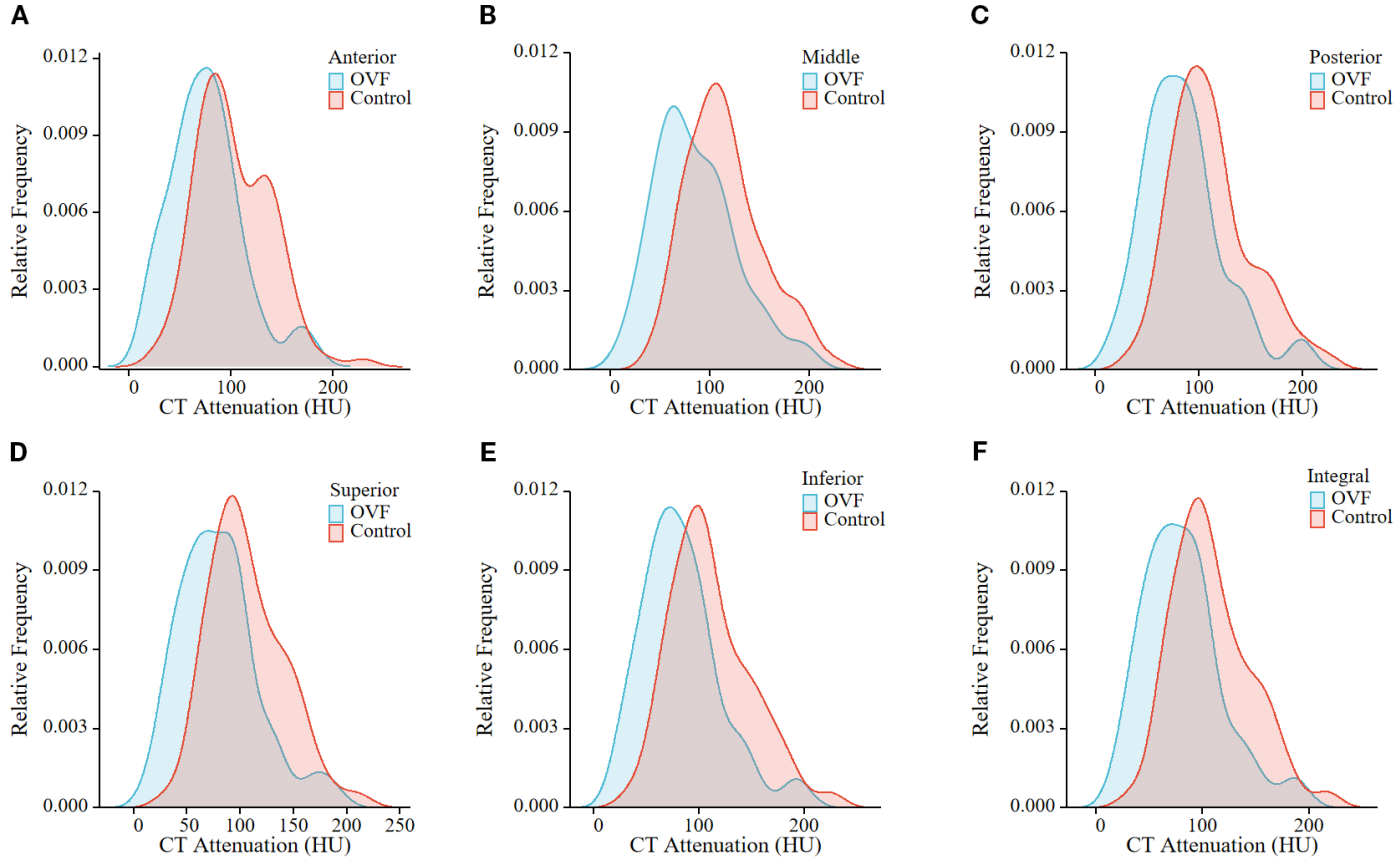
Relative frequency polygon shows CT attenuation of the anterior region **(A)**, middle region **(B)**, posterior region **(C)**, superior region **(D)**, and inferior region **(E)**, and total vertebrae **(F)** of L2 in the individuals with OVF and the controls. (OVF, osteoporotic vertebral fracture; HU, Hounsfield Units).

Among the morphological parameters analyzed, both the middle and posterior heights of the L1 vertebrae were significantly lower in the OVF group compared to the control group (B-H adjusted p= 0.001 and p= 0.002, respectively). No significant differences were observed in other morphological parameters of the L1 vertebrae or in any morphological parameters of the L2 vertebrae between the two groups (all B-H adjusted p> 0.05) (**Table 2**). For the HU measurement, all of the regional HU values (anterior, middle, posterior, superior, and inferior region) and the total vertebral HU values for both the L1 and L2 vertebrae were significantly lower in the OVF group compared with the control group (all B-H adjusted p< 0.001) and the details showed in **Table 2**; **Figures 3**, **4**.

**Table 2:** Comparison of the morphological parameters, CT Attenuation, HU values distribution and heterogeneity of L1 and L2 vertebrae between the OVF group and the control group.

Characteristics	L1 vertebrae			B-H adjusted P value	L2 vertebrae			B-H adjusted P value
	OVF (n= 54)	Control (n= 108)	Unadjusted P value		OVF (n= 54)	Control (n= 108)	Unadjusted P value	
Morphology								
Anterior Height (mm)	22.1 ± 2.0	22.6 ± 1.6	0.133	0.206	23.7 ± 1.8	23.7 ± 1.9	0.939	0.939
Middle Height (mm)	20.5 ± 1.8	21.5 ± 1.7	**< 0.001**	**0.001**	21.5 ± 2.7	21.9 ± 2.3	0.401	0.487
Posterior Height (mm)	22.8 ± 2.0	24.0 ± 1.7	**< 0.001**	**0.002**	23.8 ± 2.0	24.3 ± 1.9	0.107	0.144
A-P width (mm)	26.0 ± 2.9	28.6 ± 25.3	0.454	0.593	28.8 ± 4.1	27.5 ± 3.1	0.053	0.100
R-L width (mm)	34.6 ± 2.6	34.6 ± 3.2	0.997	0.997	38.0 ± 8.2	36.3 ± 3.7	0.067	0.114
CT Attenuation (HU)								
Anterior	76.6 ± 35.9	102.8 ± 36.1	**< 0.001**	**< 0.001**	63.3 ± 34.7	95.7 ± 36.5	**< 0.001**	**< 0.001**
Middle	86.2 ± 40.6	115.7 ± 38.3	**< 0.001**	**< 0.001**	77.2 ± 38.9	109.1 ± 37.6	**< 0.001**	**< 0.001**
Posterior	85.1 ± 38.1	111.9 ± 38.7	**< 0.001**	**< 0.001**	81.8 ± 40.0	113.4 ± 40.0	**< 0.001**	**< 0.001**
Superior	80.2 ± 37.0	106.6 ± 35.3	**< 0.001**	**< 0.001**	70.6 ± 36.6	101.2 ± 35.2	**< 0.001**	**< 0.001**
Inferior	83.6 ± 37.2	111.5 ± 38.4	**< 0.001**	**< 0.001**	76.3 ± 37.4	109.7 ± 39.5	**< 0.001**	**< 0.001**
Total	81.9 ± 36.9	109.0 ± 36.6	**< 0.001**	**< 0.001**	73.5 ± 36.7	105.5 ± 37.1	**< 0.001**	**< 0.001**
Distribution								
Anterior/Middle	0.91 ± 0.20	0.89 ± 0.14	0.583	0.682	0.81 ± 0.21	0.87 ± 0.14	0.051	0.077
Anterior/Posterior	0.92 ± 0.25	0.93 ± 0.15	0.980	0.891	0.67 ± 0.78	0.84 ± 0.15	**0.007**	0.144
Middle/Posterior	1.01 ± 0.19	1.05 ± 0.14	0.295	0.389	0.90 ± 0.76	0.97 ± 0.14	0.228	0.583
Superior/Inferior	0.96 ± 0.14	0.96 ± 0.08	0.841	0.889	0.91 ± 0.21	0.93 ± 0.09	0.805	0.598
Heterogeneity								
IQR	20.5 ± 9.1	23.9 ± 11.3	0.061	0.086	24.2 ± 12.3	27.7 ± 13.5	0.070	0.144
CV	0.20 ± 0.12	0.15 ± 0.06	**0.023**	**0.014**	0.31 ± 0.29	0.18 ± 0.07	**< 0.001**	**0.005**

(SD, standard deviation; OVF, osteoporotic vertebral fracture; A-P, anterior-posterior, R-L, right-left, IQR, interquartile range; CV, coefficient of variation; HU, Hounsfield Units; B-H, Benjamini-Hochberg method). All measurements were presented as mean ± SD. P value in bold indicated statistical significance.

### Spatial distribution and heterogeneity in vertebral CT attenuation

**Figure 5:**
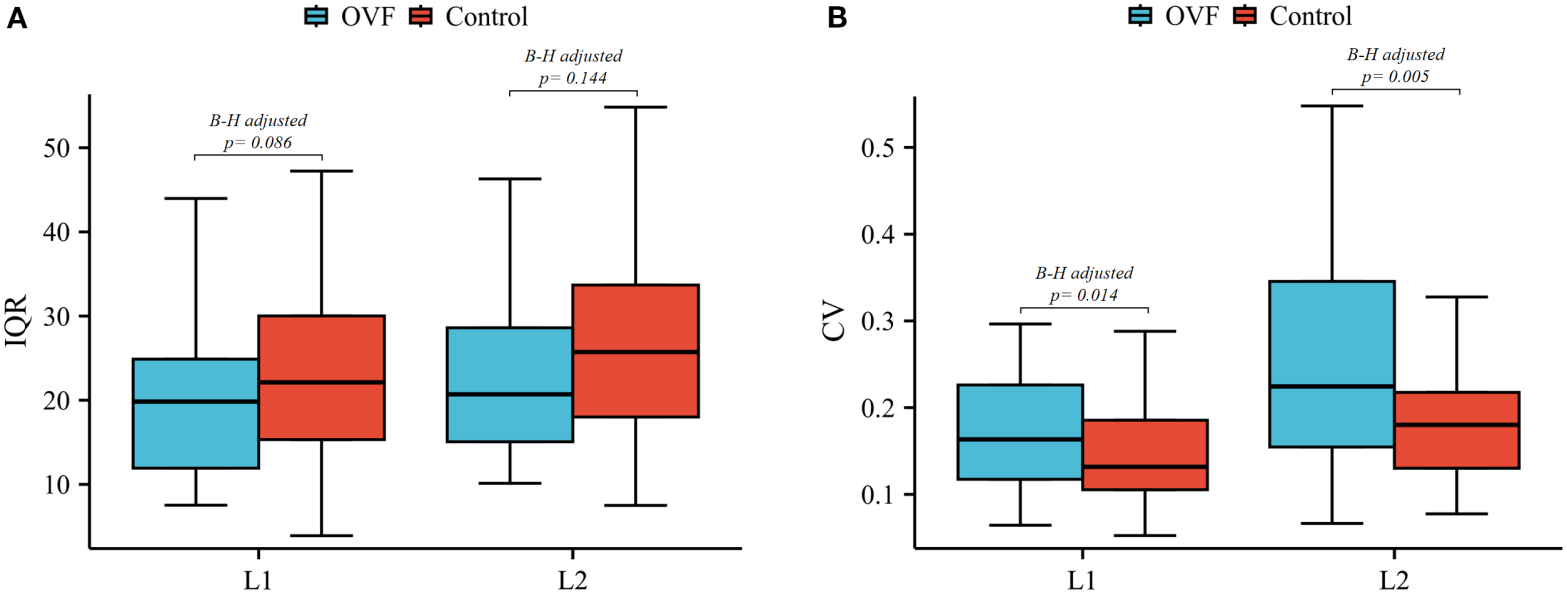
Box plots of the IQR **(A)** and CV **(B)** of L1 and L2 vertebrae between the OVF and control groups. (OVF, osteoporotic vertebral fracture; IQR, interquartile range; CV, coefficient of variation; B-H, Benjamini-Hochberg method).

The anterior/middle and anterior/posterior HU ratio of the L2 vertebrae were lower in the OVF group compared to the control group (anterior/middle: 0.81 ± 0.21 versus 0.87 ± 0.14, p= 0.051; anterior/posterior: 0.67 ± 0.78 versus 0.84 ± 0.15, p= 0.007), however, the difference did not reach statistical significance after B-H correction (both B-H adjusted p> 0.05) (**Table 2**). Further, patients with OVF exhibited higher CV in the L1 vertebrae (0.20 ± 0.12 versus 0.15 ± 0.06, B-H adjusted p= 0.014) and the L2 vertebrae (0.31 ± 0.29 versus 0.18 ± 0.07, B-H adjusted p= 0.005) compared to control subjects. The IQRs of the L1 vertebrae (20.5 ± 9.1 versus 23.9 ± 11.3 HU, B-H adjusted p= 0.086) and L2 vertebrae (24.2 ± 12.3 versus 27.7 ± 13.5 HU, B-H adjusted p= 0.144) were similar between the OVF group and control subjects (**Table 2**; **Figure 5**).

OVF was associated with vertebral CT attenuation and some measures of spatial distribution and intravetebral heterogeneity. The HU value of the L1 vertebrae (per 5 HU increment) demonstrated a negative association with OVF (OR 0.895; 95% CI, 0.847 - 0.945; p< 0.001). Similarly, the HU value of the L2 vertebrae (per 5 HU increment) were inversely related to OVF (OR 0.880; 95% CI, 0.832 - 0.930; p< 0.001). Additionally, the HU ratios of anterior/middle (OR 0.084; 95% CI, 0.011 - 0.666; p= 0.019) and anterior/posterior (OR 0.098; 95% CI, 0.014 - 0.674; p= 0.018) of the L2 vertebrae showed a modest negative correlation with OVF, but not for L1 vertebrae. Furthermore, increased CV (per 10% increment) of both the L1 vertebrae (OR 1.859; 95% CI, 1.244 - 2.780; p= 0.002) and L2 vertebrae (OR 2.045; 95% CI, 1.401 - 2.987; p< 0.001) were associated with increased odds of OVF. The multivariate logistic regression model showed that this association persisted after adjustment for BMI, morphological parameters of the vertebrae, vertebral HU value, and T-score of the lumbar spine (L1: adjusted OR 2.845; 95% CI, 1.076 - 7.524; p= 0.035 and L2: adjusted OR 2.944; 95% CI, 1.246 - 6.955; p= 0.014). No association was found between IQR and OVF in L1 (p= 0.053) and L2 (p= 0.112) vertebrae (**Table 3**; **Table 4**).

**Table 3:** ORs (95% CIs) for association between OVF and morphology and CT attenuation of L1 vertebra, distribution and heterogeneity in trabecular HU values of L1 vertebra, BMI, and BMD (T-score) of lumbar spine.

Characteristics	Univariate analysis		Multivariate analysis	
	Odds ratio (95% CI)	P value	Odds ratio (95% CI)	P value
Morphology				
Anterior Height	0.858 (0.711 – 1.034)	0.108	1.306 (0.948 – 1.799)	0.103
Middle Height	0.700 (0.568 – 0.863)	**0.001**	0.921 (0.652 – 1.301)	0.640
Posterior Height	0.714 (0.590 – 0.864)	**0.001**	0.687 (0.486 – 0.971)	**0.034**
A-P width	0.976 (0.878 – 1.086)	0.659		
R-L width	1.000 (0.897 – 1.114)	0.997		
CT Attenuation (Increased per 5 HU)	0.895 (0.847 – 0.945)	**< 0.001**	1.047 (0.935 – 1.172)	0.427
Distribution				
Anterior/Middle	1.787 (0.255 – 12.539)	0.559		
Anterior/Posterior	0.808 (0.142 – 4.582)	0.810		
Middle/Posterior	0.267 (0.032 – 2.231)	0.223		
Superior/Inferior	0.597 (0.026 – 13.554)	0.746		
Heterogeneity				
IQR	0.967 (0.934 – 1.000)	0.053	0.952 (0.888 – 1.020)	0.163
CV (Increased per 10%)	1.859 (1.244 – 2.780)	**0.002**	2.845 (1.076 – 7.524)	**0.035**
BMI	0.949 (0.832 – 1.084)	0.441		
BMD (T-Score)	0.528 (0.399 – 0.699)	**< 0.001**	0.649 (0.442 – 0.951)	**0.027**

(OR, odds ratios; OVF, osteoporotic vertebral fracture; HU, Hounsfield Units; A-P, anterior-posterior, R-L, right-left; IQR, interquartile range; CV, coefficient of variation; BMI, body mass index; BMD, bone mineral density). P value in bold indicated statistical significance.

**Table 4:** ORs (95% CIs) for association between OVF and morphology and CT attenuation of L2 vertebra, distribution and heterogeneity in trabecular HU values of L2 vertebra, BMI, and BMD (T-score) of lumbar spine.

Characteristics	Univariate analysis		Multivariate analysis	
	Odds ratio (95% CI)	P value	Odds ratio (95% CI)	P value
Morphology				
Anterior Height	0.993 (0.834 – 1.182)	0.939		
Middle Height	0.938 (0.809 – 1.089)	0.403		
Posterior Height	0.868 (0.730 – 1.032)	0.108	0.954 (0.774 – 1.176)	0.657
A-P width	1.105 (1.006 – 1.214)	**0.037**	1.147 (1.007 – 1.305)	**0.039**
R-L width	1.061 (0.988 – 1.139)	0.105	1.025 (0.944 – 1.113)	0.553
CT Attenuation (Increased per 5 HU)	0.880 (0.832 – 0.930)	**< 0.001**	1.093 (0.965 – 1.238)	0.161
Distribution				
Anterior/Middle	0.084 (0.011 – 0.666)	**0.019**	1.047 (0.040 – 27.736)	0.978
Anterior/Posterior	0.098 (0.014 – 0.674)	**0.018**	0.730 (0.033 – 16.304)	0.843
Middle/Posterior	0.720 (0.336 – 1.542)	0.397		
Superior/Inferior	0.421 (0.042 – 4.198)	0.461		
Heterogeneity				
IQR	0.978 (0.952 – 1.005)	0.112	0.945 (0.891 – 1.003)	0.061
CV (Increased per 10%)	2.045 (1.401 – 2.987)	**< 0.001**	2.944 (1.246 – 6.955)	**0.014**
BMI	0.949 (0.832 – 1.084)	0.441		
BMD (T-Score)	0.528 (0.399 – 0.699)	**< 0.001**	0.558 (0.365 – 0.854)	**0.007**

(OR, odds ratios; OVF, osteoporotic vertebral fracture; HU, Hounsfield Units; A-P, anterior-posterior, R-L, right-left; IQR, interquartile range; CV, coefficient of variation; BMI, body mass index; BMD, bone mineral density). P value in bold indicated statistical significance.

### Performance of vertebral CT attenuation in predicting OVF

**Figure 6:**
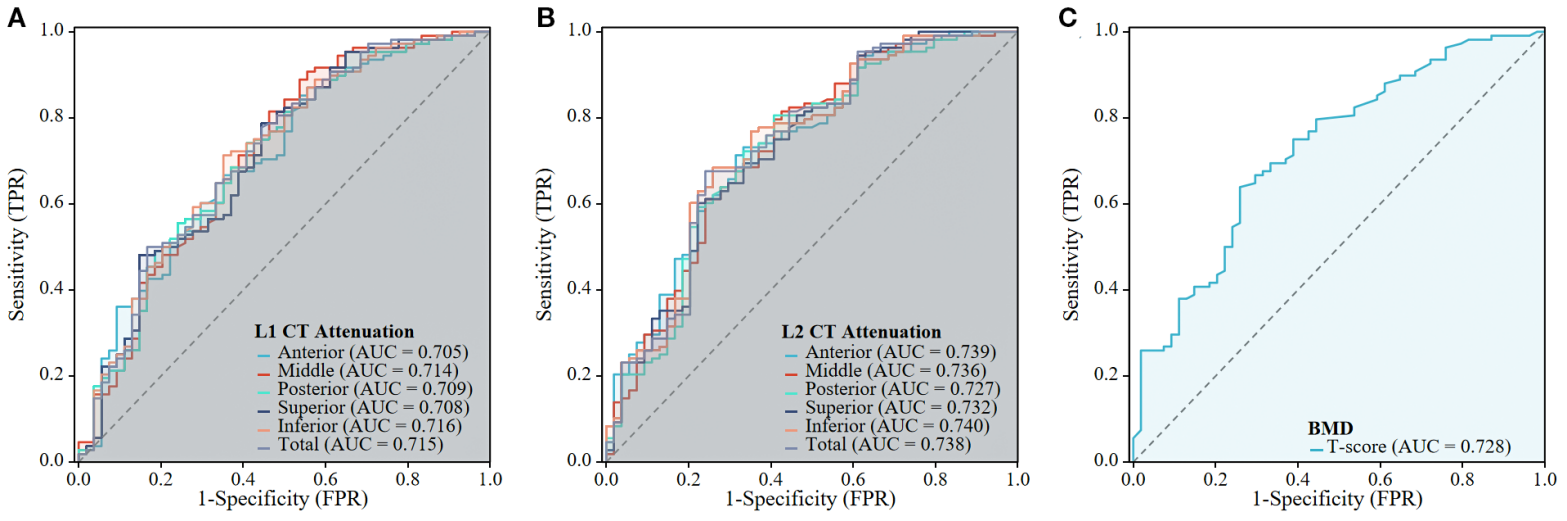
The predictive performance of the CT Attenuation of L1 and L2 vertebrae, and BMD of lumbar spine for OVF. The ROC curves are shown for the L1 CT Attenuation **(A)**, L1 CT Attenuation **(B)**, and BMD of lumbar spine **(C)**.

The ROC curve analyses, which determine the capability of various regional HU values and total vertebral HU values to differentiate patients with OVF from controls, are shown in **Table 5**; **Figure 6**. The AUC for the total vertebral HU value of the L1 and L2 vertebrae in predicting OVF were 0.715 and 0.738, respectively. Notably, the AUC for the total vertebral HU value of L2 vertebrae was higher than that for the T-score of the lumbar spine (AUC = 0.728), but this difference was not statistically significant (DeLong test p= 0.776). Among distinct intravertebral regions, the AUC of the inferior regions was marginally higher than that of other regions for both L1 (AUC = 0.716) and L2 (AUC = 0.740) vertebrae; however, these intravertebral regional differences did not reach statistical significance (DeLong test, all p > 0.05).

**Table 5:** Performance of the regional and total vertebral CT attenuation of L1 and L2 and BMD of lumbar spine for predicting OVF.

Variable	Cut-off value	AUC (95% CI)	Sensitivity	Specificity	PPV	NPV	Accuracy
L1 Attenuation							
Anterior	85 HU	0.705 (0.618 - 0.792)	0.648	0.667	0.796	0.486	0.654
Middle	80 HU	0.714 (0.625 - 0.802)	0.815	0.537	0.779	0.592	0.722
Posterior	87 HU	0.709 (0.621 - 0.796)	0.741	0.593	0.784	0.533	0.691
Superior	78 HU	0.708 (0.620 - 0.796)	0.787	0.556	0.780	0.566	0.710
Inferior	90 HU	0.716 (0.630 - 0.803)	0.713	0.648	0.802	0.530	0.691
Total	80 HU	0.715 (0.629 - 0.802)	0.778	0.556	0.778	0.556	0.704
L2 Attenuation							
Anterior	70 HU	0.739 (0.656 - 0.821)	0.731	0.667	0.814	0.554	0.710
Middle	75 HU	0.736 (0.651 - 0.821)	0.815	0.574	0.793	0.608	0.735
Posterior	83 HU	0.727 (0.641 - 0.814)	0.806	0.592	0.798	0.604	0.735
Superior	89 HU	0.732 (0.647 - 0.817)	0.602	0.778	0.844	0.494	0.660
Inferior	88 HU	0.740 (0.655 - 0.824)	0.685	0.741	0.841	0.541	0.704
Total	87 HU	0.738 (0.653 - 0.823)	0.676	0.759	0.849	0.539	0.704
BMD (T-score)	-2.5 SD	0.728 (0.646 - 0.810)	0.639	0.741	0.831	0.506	0.673

(OVF, osteoporotic vertebral fracture; BMD, bone mineral density; HU, Hounsfield Units; AUC, area under the receiver operating characteristic curve; CI, confidence interval; PPV, positive predictive value; NPV, negative predictive value).

## Discussion

This age- and sex-matched case-control study sought to systematically evaluate the association between CT attenuation (HU values) of the L1 and L2 vertebrae and OVF occurrence, with specific focus on spatial distribution and heterogeneity patterns. The results show that individuals with OVF exhibit significantly lower HU values in both the L1 and L2 vertebrae compared with control subjects. Additionally, the HU values were difference among distinct trabecular regions of the vertebrae. The inferior regions of both the L1 and L2 vertebrae demonstrated marginally superior performance in predicting OVF compared to other regions (anterior, middle, posterior, or superior regions) of each vertebra, however, these differences did not reach statistical significance. Furthermore, increased heterogeneity in HU values of the L1 and L2 vertebrae, as measured by CV, demonstrated an independent association with OVF after adjusting for BMI, morphological parameters of the vertebrae, vertebral HU value, and lumbar spine T-scores. These findings suggest that regional variations and spatial heterogeneity in CT attenuation within the vertebrae may serve as critical structural determinants of spinal bone fragility.

Recent clinical studies (9, 10, 12, 22–24) have demonstrated that vertebral trabecular HU values obtained from routine chest, abdomen, and/or lumbar spine CT scans can serve as an easily accessible and clinically promising biomarker for opportunistic screening of osteoporosis and the risk of fragility fractures. The positive correlation between HU values and BMD as measured by DXA has been established, with correlation coefficients ranging from 0.693 to 0.786 (24, 25). This relationship is expected, as a decrease in HU values corresponds to a reduction in BMD, which can lead to fragility fractures. Patients with fragility vertebral fracture generally present with lower HU values compare to the controls, and HU values have demonstrated strong predictive capability for vertebral fractures (12, 13, 23). Consistent with these findings, our study observed that individuals with OVF presented with approximately 25% lower HU values at L1 vertebra (81.9 ± 36.9 versus 109.0 ± 36.6 HU) and 30% at L2 vertebra (73.5 ± 36.7 versus 105.5 ± 37.1 HU) compared to the controls. Furthermore, the performance (AUC = 0.738) of HU value at the L2 vertebra, with a threshold of 87 HU for identifying OVF, was slightly higher than that of the lumbar spine T-score (AUC = 0.728), but this difference did not achieve statistical significance. In a similar comparable study, Bo Zhang et al (23) reported that the mean HU values of the L1-4 vertebrae exhibited superior predictive efficacy for thoracolumbar fragility fractures, with an AUC of 0.863 at an 88 HU threshold, compared to DXA-derived BMD measurements (AUC = 0.813).

However, previous studies indicated that vertebral bodies exhibit inherent variation among different regions in both BMD and trabecular architecture (16, 20, 26). Region-specific microstructural parameters, such as regional BMD or bone volume fraction (BV/TV), are better associated with vertebral mechanical strength compared to global vertebral analysis (18). Longitudinal changes in BMD also differ among various intravertebral regions. Hugo Giambini et al (27) reported that the anterior BMD of lumbar vertebrae decreased more significantly than the posterior BMD over a six-year follow-up period (Δ anterior: ~18%; Δ posterior: ~13%). This anterior-posterior gradient in bone loss progression may partially explain the clinical predominance of wedge-shaped vertebral fractures. Consequently, the average measurements of the whole vertebral characteristics (e.g., HU values or BMD) may be limited the predictive capacity for assessing individual fracture risk in clinical practice.

In our study, beyond the primary contribution of lower absolute HU values, the results highlight that regional variations and spatial heterogeneity of vertebral trabecular attenuation are critical factors significantly associated with OVF. We defined five distinct anatomical regions (anterior, middle, posterior, superior, and inferior) within the L1 and L2 vertebrae using three-dimensional volumetric method to investigate the predictive capacity of region-specific HU values for OVF. The results revealed slight variation in AUCs across different anatomical regions (L1: AUC = 0.705-0.716; L2: AUC = 0.727-0.740). The inferior regions have marginally superior predictive performance relative to other regions; however, these differences did not reach statistical significance when compared to whole-vertebra measurements (total vertebral HU values). We further investigated the association between the spatial distribution of HU values and OVF. Our findings indicated that a decreased regional HU ratio of anterior/middle and anterior/posterior in the L2 vertebra may be associated with an increased odds of OVF (anterior/middle: OR = 0.084 [0.011 - 0.666]; anterior/posterior: OR = 0.098 [0.014 - 0.674]). However, these association were not independent of BMI, morphological parameters of the vertebrae, and T-score of lumbar spine. These regional variations and spatial distribution patterns suggest that while regional HU variations may reflect localized biomechanical vulnerabilities, their incremental predictive value over conventional entire-vertebra assessments requires further validation in larger cohorts.

Quantification of spatial heterogeneity of trabecular density and microstructure within vertebral bodies, as assessed by QCT or micro-CT, has revealed significant biomechanical correlations with vertebral strength. However, the existing evidence exhibits paradoxical results regarding the directional relationship between trabecular heterogeneity and vertebral biomechanics. While certain studies have identified positive associations between increased heterogeneity and critical mechanical parameters such as strength, stiffness, and toughness (19, 28), other investigations have reported inverse correlations (29–31). These conflicting observations may be attributed to methodological variations inherent in study designs, particularly in trabecular density sampling protocols, non-uniform mechanical loading modes (axial versus eccentric), anatomical variations in specimen selection (differences in vertebral level), and discrepancies in structural complexity between isolated vertebrae and multi-segment spine preparations. Our research indicated that patients with OVF demonstrate higher spatial heterogeneity (quantified by CV) in HU values within the L1 and L2 vertebrae compared to control subjects. Moreover, the elevated CV was independently associated with OVF after adjusting for BMI, morphological parameters of the vertebrae, vertebral HU value, and lumbar spine T-scores. These finds lending support to an inverse relationship between vertebral trabecular spatial heterogeneity and vertebral strength.

## Limitations

This study has several limitations. Firstly, although quantitative CT (QCT) derived volumetric BMD would have allowed for a more accurate assessment of bone density and fracture risk (11, 32), its measurement requires a dedicated calibration phantom for simultaneous or asynchronous calibration. This requirement limits its application in daily clinical practice, particularly in retrospective studies and phantom-limited institutions. Given the retrospective nature of our lumbar spine CT data collection, we utilized volumetric HU values instead of volumetric BMD. This approach is straightforward to implement and can be applied directly within the Picture Archiving and Communication System (PACS) or free available software (ie, 3D slicer), offering broader clinical applicability. Secondly, our analysis focused on HU values at the L1 and L2 vertebrae, as these levels are the most common sites for OVF and are readily accessible in chest, abdominal, or lumbar CT scans. However, other vertebral levels, such as L3-L5, may demonstrate different patterns of mineral density changes due to variations in biomechanical loading and age-related degeneration. Consequently, future research should aim to explore the relationship between CT-derived HU measurements and fracture risk across the entire lumbar spine, including the lower vertebral levels, to develop a more comprehensive understanding of regional differences in bone density and fracture susceptibility. Finally, it is crucial to recognize that the retrospective nature of our study precluded the assessment of longitudinal changes in regional variations, distribution patterns, and heterogeneity of HU values within the vertebral bodies. Future research utilizing a prospective design may elucidate the temporal evolution of these parameters and their association with fracture risk.

## Conclusions

This case-control study demonstrated that reduced regional and total vertebral HU values, and increased spatial heterogeneity in the L1 and L2 vertebrae were associated with higher odds of OVF. These findings contribute novel insights into the structural determinants of vertebral bone fragility and serve as valuable references for evaluating vertebral fracture risk in clinical practice.

## Data Availability

The raw data supporting the conclusions of this article will be made available by the authors, without undue reservation.
